# Supplementary material for: Dual-color optical activation and suppression of neurons with high temporal precision
Source: eLife. 2025 May 13;12:RP90327. doi: 10.7554/eLife.90327 (PMC12074635; doi:10.7554/eLife.90327)
Supplement: Supplementary file 1. — For ZipACR(151V), position 151 of ZipACR is valine and encoded by the codon GTC and the sequence of IvfChrimson-citrine starts at polypeptide sequence position 409. Note that the flexible linkers and amino acids introduced by the digestion sites are not annotated. [file elife-90327-supp1.docx]

**ZipACR**

**151T**

**KV2.1 signalling**

**2A**

**ChIEF/ReaChR N-terminus**

**vfChrimson**

**Trafficking sequence**

**citrine**

**KV2.1 signalling**

1 M A A L H Q A L E E L G Q G A S R H L L 20

1 ATGGCCGCCCTCCATCAGGCTCTGGAGGAGCTGGGACAGGGAGCCAGCAGGCACCTCCTG 60

21 Y R A P E E M V Y G K W V E A N P R C I 40

61 TACAGAGCCCCCGAGGAGATGGTGTATGGCAAGTGGGTGGAGGCCAACCCTAGGTGCATC 120

41 E L H D H S D Y V Y V F Q L C F A V V C 60

121 GAACTGCACGACCACTCCGACTACGTGTACGTGTTCCAGCTGTGCTTCGCCGTGGTGTGC 180

61 A C Q V I F M F T R A P N V G W E A I Y 80

181 GCCtGCCAGGTGATCTTCATGTTCACCAGGGCCCCCAACGTGGGCTGGGAAGCCATCTAT 240

81 L P L A E V V T Y S I A A N G E G V L R 100

241 CTGCCCCTGGCCGAGGTCGTGACCTACAGCATTGCCGCCAACGGAGAGGGCGTGCTGAGG 300

101 M A D G R Y F N F A K L A G W A V C C P 120

301 ATGGCCGACGGCAGGTACTTCAATTTCGCCAAACTGGCCGGATGGGCCGTGTGCTGCCCC 360

121 I M L I Q I G G M A Q I K Y R T I P L N 140

361 ATTATGCTCATCCAGATCGGAGGCATGGCCCAGATCAAGTACAGGACCATCCCCCTGAAC 420

141 N V V L A A S L N R T I F G M A S A I T 160

421 AACGTGGTGCTGGCCGCTAGCCTGAATAGGACCATCTTCGGCATGGCCAGCGCCATCACA 480

161 A S D P A R W G F Y F C A W I C Y L T E 180

481 GCCTCCGACCCTGCTAGATGGGGCTTTTACTTCTGCGCCTGGATCTGTTACCTGACCGAG 540

181 V G I T L T I M A V A I S D F S K I K T 200

541 GTCGGCATCACCCTGACCATCATGGCCGTGGCCATCAGCGACTTCAGCAAGATCAAGACA 600

201 E L G Q W V V G R I Q T M R I I F L V A 220

601 GAGCTCGGCCAGTGGGTGGTGGGCCGTATTCAAACCATGAGGATCATCTTTCTGGTCGCC 660

221 W T S F P V V W V L G Y T G F C V I H E 240

661 TGGACCAGCTTCCCCGTCGTGTGGGTGCTGGGCTACACCGGATTCTGCGTGATCCACGAG 720

241 D Y I A L L Y L F A D L L S K N T W G V 260

721 GACTACATCGCCCTGCTGTATCTGTTCGCCGACCTGCTCTCCAAGAACACCTGGGGAGTG 780

261 M M W H T T W V K L N G K W D R E F A A 280

781 ATGATGTGGCATACCACCTGGGTGAAACTCAACGGCAAATGGGACAGGGAATTTGCCGCC 840

281 A G G H E A L K K A L E Q D V E I G A G 300

841 GCCGGAGGACACGAGGCTCTGAAGAAGGCCCTGGAGCAGGACGTGGAAATCGGCGCCGGC 900

301 E K N Q N Q L T A R Q A A A Q S Q P I L 320

901 GAGAAGAACCAGAACCAGCTGACCGCCAGGCAGGCGGCCGCgCAGAGCCAGCCCATCTTG 960

321 N T K E M A P Q S K P P E E L E M S S M 340

961 AATACCAAGGAGATGGCCCCCCAGAGCAAGCCCCCCGAGGAGCTGGAGATGAGCAGCATG 1020

341 P S P V A P L P A R T E G V I D M R S M 360

1021 CCCAGCCCCGTGGCCCCCCTGCCCGCCAGAACCGAGGGCGTGATAGATATGAGGTCTATG 1080

361 S S I D S F I S C A T D F P E A T R F A 380

1081 AGCAGCATCGACAGCTTCATCTCATGTGCAACTGACTTCCCCGAGGCCACCAGATTCgct 1140

381 S G G G S G G G E G R G S L L T C G D V 400

1141 agcggaggtggatctggtggcggtgagggcagaggaagtcttctaacatgcggtgacgtg 1200

401 E E N P G P R T M V S R R P W L L A L A 420

1201 gaggagaatcccggccctcgtacgATGGTGAGCAGAAGACCCTGGCTGCTGGCCCTGGCC 1260

421 L A V A L A A G S A G A S T G S D A T V 440

1261 CTGGCCGTGGCCCTGGCCGCCGGCAGCGCCGGCGCCAGCACCGGCAGCGACGCCACCGTG 1320

441 P V A T Q D G P D Y V F H R A H E R M L 460

1321 CCCGTGGCCACCCAGGACGGCCCCGACTACGTGTTCCACAGAGCCCACGAGAGAATGCTG 1380

461 F Q T S Y T L E N N G S V I C I P N N G 480

1381 TTCCAGACCAGCTACACCCTGGAGAACAACGGCAGCGTGATCTGCATCCCCAACAACGGC 1440

481 Q C F C L A W L. L H S R G T P G E K I G 500

1441 CAGTGCTTCTGCCTGGCCTGGCTGctgcacagcagaggcacaccaggagaaaagatcggc 1500

501 A Q V C Q W I A F S I A I A L L T F Y G 520

1501 gcccaggtctgccagtggattgctttcagcatcgccatcgccctgctgacattctacggc 1560

521 F S A W K A T C G W E E V Y V C C V E V 540

1561 ttcagcgcctggaaggccacttgcggttgggaggaggtctacgtctgttgcgtcgaggtg 1620

541 L F V T L E I F K E F S S P A T V Y L S 560

1621 ctgttcgtgaccctggagatcttcaaggagttcagcagccccgccacagtgtacctgtct 1680

561 T G N H A Y C L R Y F E W L L S C P V I 580

1681 accggcaaccacgcctattgcctgcgctacttcgagtggctgctgtcttgccccgtgatc 1740

581 L I R L S N L S G L K N D Y S K R T M G 600

1741 ctgatcCGCctgagcaacctgagcggcctgaagaacgactacagcaagcggaccatgggc 1800

601 L I V S C V G M I V F G M A A G L A T D 620

1801 ctgatcgtgtcttgcgtgggaatgatcgtgttcggcatggccgcaggactggctaccgat 1860

621 W L K W L L Y I V S C I Y G G Y M Y F Q 640

1861 tggctcaagtggctgctgtatatcgtgtcttgcatctacggcggctacatgtacttccag 1920

641 A A K C Y V E A N H S V P K G H C R M V 660

1921 gccgccaagtgctacgtggaagccaaccacagcgtgcctaaaggccattgccgcatggtc 1980

661 V K L M A Y A F F A S W G M Y P I L W A 680

1981 gtgaagctgatggcctacgctttcttcgcctcttggggcatgtacccaatcctctgggca 2040

681 V G P E G L L K L S P Y A N S I G H S I 700

2041 gtgggaccagaaggactgctgaagctgagcccttacgccaacagcatcggccacagcatc 2100

701 C D I I A K E F W T F L A H H L R I K I 720

2101 tgcgacatcatcgccaaggagttttggaccttcctggcccaccacctgaggatcaagatc 2160

721 H E H I L I H G D I R K T T K M E I G G 740

2161 cacgagcacatcctgatccacggcgacatccggaagaccaccaagatggagatcggaggc 2220

741 E E V E V E E F V E E. E D E D T V L E K 760

2221 gaggaggtggaagtggaagagttcgtggaggaggaggacgaggacacagtgCTCGAGaag 2280

761 S R I T S E G E Y I P L D Q I D I N V M 780

2281 agcaggatcaccagcgagggcgagtacatccccctggaccagatcgacatcaacgtgatg 2340

781 V S K G E E L F T G V V P I L V E L D G 800

2341 gtgagcaagggcgaggagctgttcaccggggtggtgcccatcctggtcgagctggacggc 2400

801 D V N G H K F S V S G E G E G D A T Y G 820

2401 gacgtaaacggccacaagttcagcgtgtccggcgagggcgagggcgatgccacctacggc 2460

821 K L T L K F I C T T G K L P V P W P T L 840

2461 aagctgaccctgaagttcatctgcaccaccggcaagctgcccgtgccctggcccaccctc 2520

841 V T T F G Y G L M C F A R Y P D H M K Q 860

2521 gtgaccaccttcggctacggcctgatgtgcttcgcccgctaccccgaccacatgaagcag 2580

861 H D F F K S A M P E G Y V Q E R T I F F 880

2581 cacgacttcttcaagtccgccatgcccgaaggctacgtccaggagcgcaccatcttcttc 2640

881 K D D G N Y K T R A E V K F E G D T L V 900

2641 aaggacgacggcaactacaagacccgcgccgaggtgaagttcgagggcgacaccctggtg 2700

901 N R I E L K G I D F K E D G N I L G H K 920

2701 aaccgcatcgagctgaagggcatcgacttcaaggaggacggcaacatcctggggcacaag 2760

921 L E Y N Y N S H N V Y I M A D K Q K N G 940

2761 ctggagtacaactacaacagccacaacgtctatatcatggccgacaagcagaagaacggc 2820

941 I K V N F K I R H N I E D G S V Q L A D 960

2821 atcaaggtgaacttcaagatccgccacaacatcgaggacggcagcgtgcagctcgccgac 2880

961 H Y Q Q N T P I G D G P V L L P D N H Y 980

2881 cactaccagcagaacacccccatcggcgacggccccgtgctgctgcccgacaaccactac 2940

981 L S Y Q S A L S K D P N E K R D H M V L 1000

2941 ctgagctaccagtccgccctgagcaaagaccccaacgagaagcgcgatcacatggtcctg 3000

1001 L E F V T A A G I T L G M D E L Y K Q S 1020

3001 ctggagttcgtgaccgccgccgggatcactctcggcatggacgagctgtacaagCAGTCC 3060

1021 Q P I L N T K E M A P Q S K P P E E L E 1040

3061 CAACCCATACTGAACACGAAGGAAATGGCTCCTCAAAGTAAACCGCCCGAAGAACTCGAa 3120

1041 M S S M P S P V A P L P A R T E G V I D 1060

3121 ATGAGTTCAATGCCATCACCTGTGGCTCCGCTTCCCGCCCGCACGGAAGGTGTCATCGAC 3180

1061 M R S M S S I D S F I S C A T D F P E A 1080

3181 ATGCGAAGCATGTCTAGTATTGACTCATTTATTAGCTGCGCCACCGATTTCCCTGAAGCT 3240

1081 T R F * 1083

3241 ACTCGGTTCTAA 3252

**Supplementary Material 1: The annotated sequence of the 2A based ZipT-IvfChr design used in this study.**
