# Supplementary material for: Dual-color optical activation and suppression of neurons with high temporal precision
Source: eLife. 2025 May 13;12:RP90327. doi: 10.7554/eLife.90327 (PMC12074635; doi:10.7554/eLife.90327)
Supplement: Supplementary file 2. [file elife-90327-supp2.docx]

| **ZipT-IvfChr** | **Animal** | **Trial number** | **AUC (Red)** | **Average AUC(Red)** | **AUC (Blue)** | **Average AUC (Blue)** | **Ratio (Blue/Red)** |
| --- | --- | --- | --- | --- | --- | --- | --- |
|  | #1 | 1 | 0.549 | 0.506 | 0.119 | 0.078 | 0.155 |
|  |  | 2 | 0.509 |  | 0.080 |  |  |
|  |  | 3 | 0.460 |  | 0.036 |  |  |
|  | #2 | 1 | 0.386 | 0.480 | 0.126 | 0.083 | 0.174 |
|  |  | 2 | 0.484 |  | 0.041 |  |  |
|  |  | 3 | 0.570 |  | 0.083 |  |  |
|  | #3 | 1 | 0.372 | 0.250 | 0.037 | 0.005 | 0.018 |
|  |  | 2 | 0.217 |  | -0.027 |  |  |
|  |  | 3 | 0.161 |  | 0.004 |  |  |
|  | #4 | 1 | 0.086 | 0.098 | 0.023 | 0.023 | 0.234 |
|  |  | 2 | 0.120 |  | 0.007 |  |  |
|  |  | 3 | 0.087 |  | 0.038 |  |  |
|  |  |  |  |  |  | **Mean** | **0.145** |
|  |  |  |  |  |  | **SEM** | **0.046** |
|  |  |  |  |  |  |  |  |
| **IvfChrimson** | **Animal** | **Trial number** | **AUC (Red)** | **Average AUC (Red)** | **AUC (Blue)** | **Average AUC (Blue)** | **Ratio (Blue/Red)** |
|  | #1 | 1 | 0.613 | 0.533 | 0.430 | 0.451 | 0.845 |
|  |  | 2 | 0.540 |  | 0.460 |  |  |
|  |  | 3 | 0.447 |  | 0.463 |  |  |
|  | #2 | 1 | 0.463 | 0.482 | 0.395 | 0.312 | 0.649 |
|  |  | 2 | 0.503 |  | 0.284 |  |  |
|  |  | 3 | 0.479 |  | 0.259 |  |  |
|  | #3 | 1 | 0.603 | 0.572 | 0.259 | 0.267 | 0.467 |
|  |  | 2 | 0.541 |  | 0.275 |  |  |
|  |  | 3 | 0.571 |  | 0.267 |  |  |
|  | #4 | 1 | 0.574 | 0.580 | 0.375 | 0.427 | 0.737 |
|  |  | 2 | 0.535 |  | 0.469 |  |  |
|  |  | 3 | 0.631 |  | 0.438 |  |  |
|  |  |  |  |  |  | **Mean** | **0.674** |
|  |  |  |  |  |  | **SEM** | **0.080** |

**Supplementary material 2: Area under the curve (AUC) for the whiskers protraction triggered by red or blue-light illumination of the facial nucleus neurons expressing ZipT-IvfChr or IvfChrimson.**
